# Surveying the local public health response to COVID-19 in Canada: Study protocol

**DOI:** 10.1371/journal.pone.0259590

**Published:** 2021-11-18

**Authors:** Charles Plante, Thilina Bandara, Lori Baugh Littlejohns, Navdeep Sandhu, Anh Pham, Cory Neudorf

**Affiliations:** 1 Johnson Shoyama Graduate School of Public Policy, University of Saskatchewan, Saskatoon, Saskatchewan, Canada; 2 Department of Community Health and Epidemiology, University of Saskatchewan, Saskatoon, Saskatchewan, Canada; Universitat Luzern, SWITZERLAND

## Abstract

**Background:**

Public health services and systems research is under-developed in Canada and this is particularly the case with respect to research on local public health unit operational functioning and capacity. The purpose of this paper is to report on a study that will collect retrospective information on the local public health response to COVID-19 throughout Canada between 2020 and 2021.

**Methods/Design:**

The goal of the study is to develop and implement a study framework that will collect retrospective information on the local public health system response to the COVID-19 pandemic in Canada. This study will involve administering a mixed-method survey to Medical Health Officers/Medical Officers of Health in every local and regional public health unit across the country, followed by a process of coding and grouping these responses in a consistent and comparable way. Coded responses will be assessed for patterns of divergent or convergent roles and approaches of local public health across the country with respect to interventions in their response to COVID-19. The Framework Method of thematic analysis will be applied to assess the qualitative answers to the open-ended questions that speak to public health policy features.

**Discussion:**

The strengths of the study protocol include the engagement of Medical Health Officers/Medical Officers of Health as research partners and a robust integrated knowledge translation approach to further public health services and systems research in Canada.

## Introduction

“Pandemics happen locally and impact globally” states the Chief Medical Officer of Health of Canada in her 2020 report that focuses on the COVID-19 pandemic and in particular, health equity concerns [1]. The report identifies that “COVID-19 outbreaks across Canada have had different effects on different populations. Effective public health measures seek to recognize and target these local contexts and regional differences” [1]. Throughout the country, local public systems are responsible for implementing and tailoring provincially and federally identified COVID-19 measures to meet the needs of individual communities. Typically led (or co-led) by Medical Health Officers/Medical Officers of Health (MHO/MOH), who have various titles across the country however, all have public health physician training. These local systems vary greatly throughout the country in their governance structure, scope of oversight and responsibility, and operating capacity. Many strain even to deploy basic public health activities during non-crisis periods. Broadly speaking, there is a paucity of research focused on local public health operations in Canada [2–4].

Although COVID-19 has recently stimulated greater interest in public health systems and services, historically public health systems and services research has been under-developed in Canada [5] and still does not extend to the local level. This paper describes the study protocol for a novel “public health policy surveillance” framework that will collect retrospective information on the local public health response to COVID-19 throughout Canada between 2020 and 2021. It will do so by administering a mixed-method survey to MHO/MOH in every local and regional public health unit across the country, and then coding and grouping these responses in a consistent and comparable way. In addition to providing additional insight into the public health response to COVID-19—What is local public health in Canada? How did it respond to the COVID-19 public health crisis? And, how have these responses varied across Canada and why?—we hope that it will also provide a foundation for a more robust public health services and systems research program in Canada in the future.

**Fig 1:**
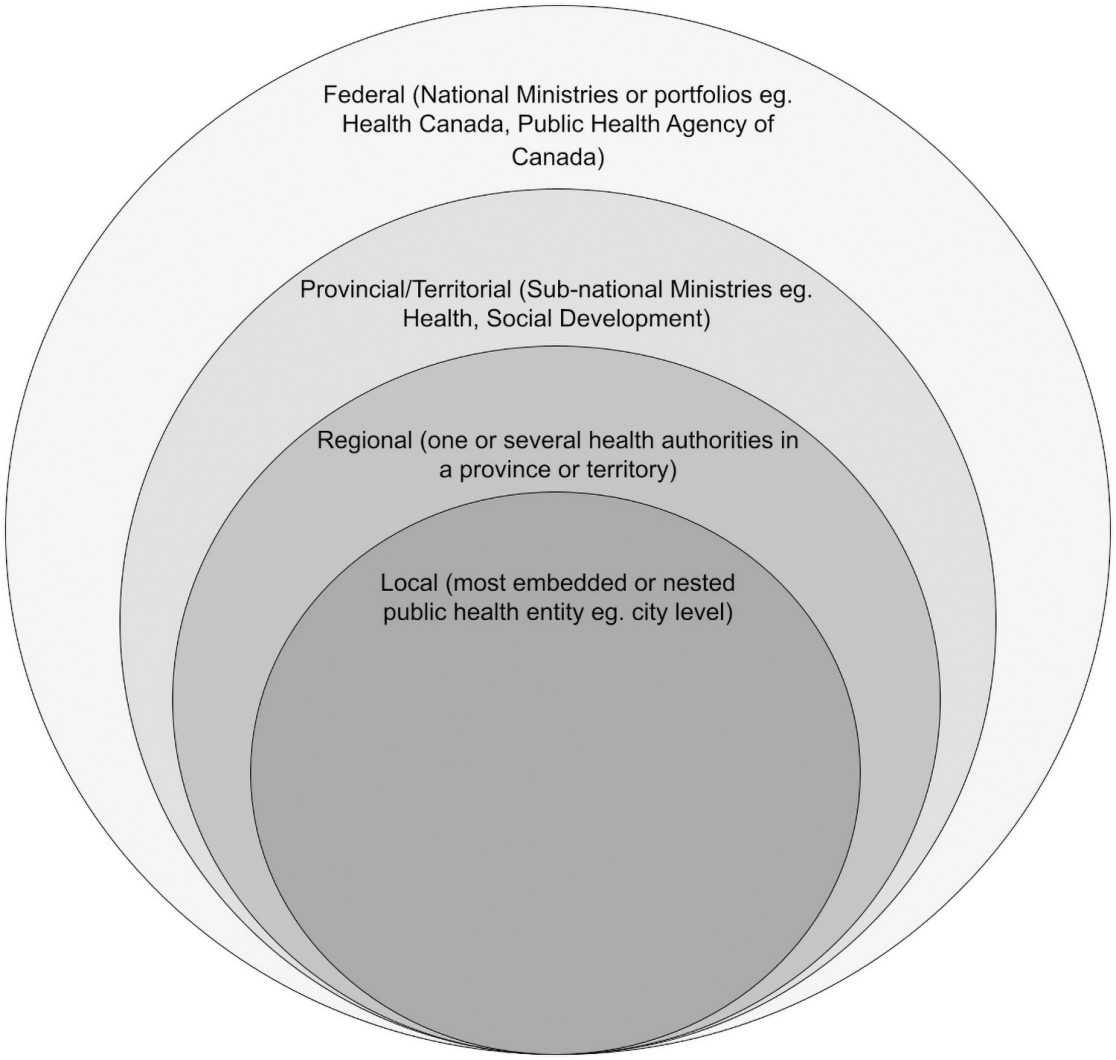
Local public health units in nested public health governance.

For this study, we define as our unit of analysis, local public health units (LPHU), which we operationalize as the lowest unit of independent (or delegated) responsibility for a defined population led (or co-led) by a qualified MHO/MOH (see Fig 1). Note that not all LPHU in Canada have direct authority for the administration of public health programs and services and so may not be immediately recognized or named as “units.” In some Canadian provinces, certain categories of public health programs and service delivery are provided by “regional” units, which we define as having jurisdictions that span multiple local units. These regional public health units (RPHU) are also headed by MHO/MOH (the numbers of local and regional health units in Canada is provided in Table 1).

**Table 1:** Number of regional public health units (RPHU) and local public health units (LPHU) in Canada.

Province/Territory	Number of Regional Public Health Units (RPHU)	Number of Local Public Health Units (LPHU)	Number of Indigenous-focused Regional and Local Public Health Units	Population
Alberta	1	5	1	4,436,258
British Columbia	5	16	1	5,153,039
Manitoba	0	5	3	1,380,935
New Brunswick	0	4	3	782,078
Newfoundland and Labrador	0	4	2	520,438
Northwest Territories	0	1	2	45,136
Nova Scotia	0	4	1	979,449
Nunavut	0	1	0	39,407
Ontario	0	34	1	14,755,211
Prince Edward Island	0	1	1	159,819
Quebec	0	18	3	8,575,944
Saskatchewan	1	4	3	1,178,832
Yukon Territory	0	1	0	42,192
Total	7	98	21	38,048,738

There is important historical support for studying health protection and communicable disease control at the LPHU level. For example, the widely-cited Naylor Report states that “The outbreak management teams and leaders of the local public health units were identified by some interviewees as those who deserve greatest credit for containing the SARS outbreak. The role assumed by most public health units was focused on front-line containment of the outbreak” [6].

### Public health policy surveillance

The motivation for this project is borne directly from the expressed need by local public health leadership in Canada to systemize their already ongoing cross-national policy and programming correspondence as they try to learn from each other’s successes and challenges and respond to the COVID-19 crisis. At present, these conversations are occurring informally, which a) do not allow time for comprehensive participation; b) are not optimized for capturing and documenting broader institutional knowledge beyond the leadership; and c) cannot be informed by more detailed information on differences in local operational capacity and community characteristics. The objective of this study is to systematically collect comparable data on local public health activities across the country that reflects the concerns and data needs of frontline decision makers, and can be used to inform evidence-based decision making and policy action to improve population health. Our approach resembles what Burris et al. [7] have described as “Public Health Policy Surveillance”, that is, “ongoing, systematic collection, analysis, interpretation and dissemination of information about a given body of public health law and policy.” However, rather than extract information on local public health operations from legal or policy documents, we will collect this information by directly surveying local MHO/MOH. This approach is required because much of the work of local public health in Canada is uncodified.

### An integrated knowledge translation approach

**Fig 2:**
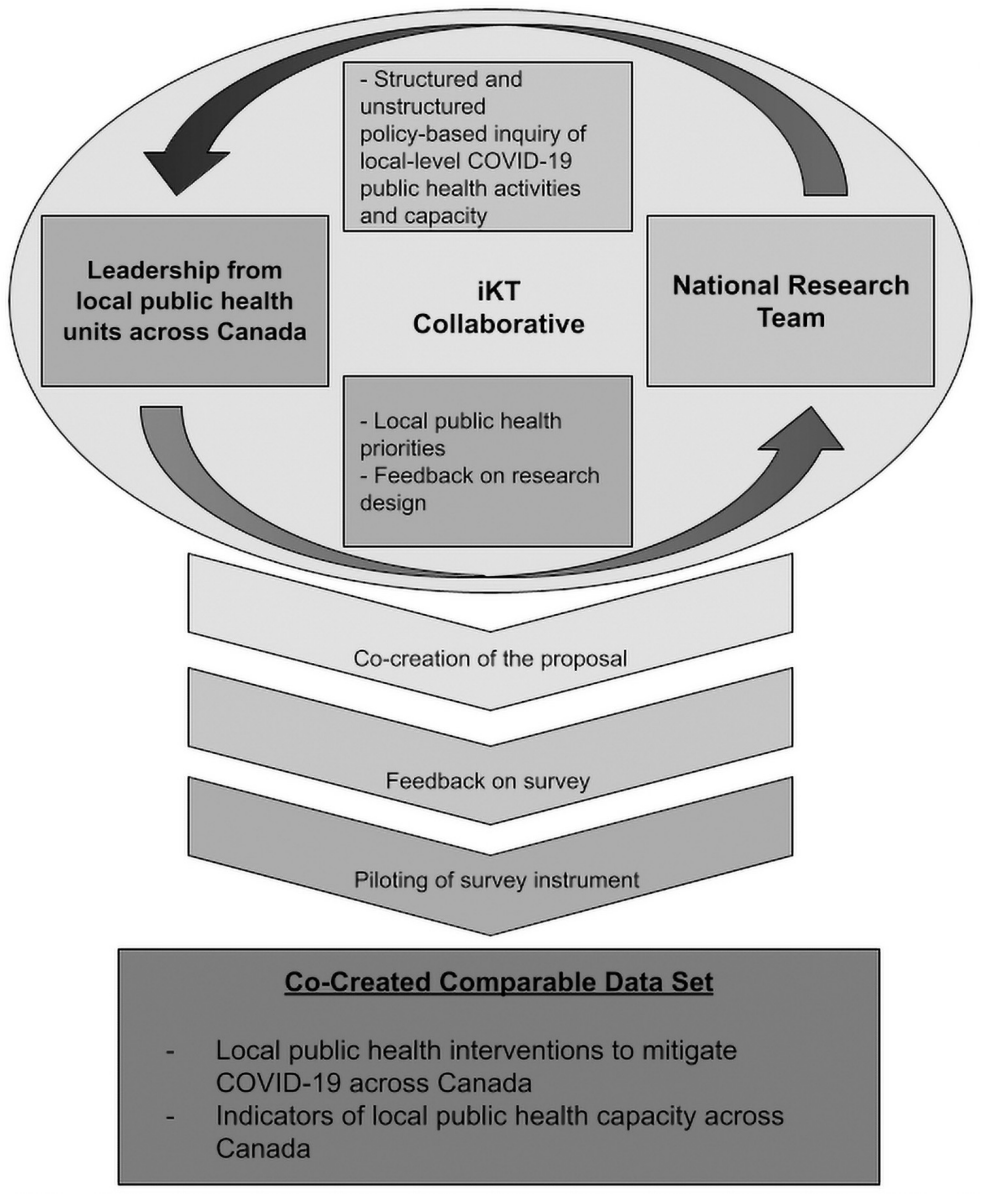
Integrate knowledge translation model.

Integrated knowledge translation is a solution to the underuse of research in policy and practice settings, which involves developing partnerships between researchers and those who apply the research into practice (see Fig 2). The relationships with knowledge users are created to enhance the relevance and uptake of research findings [8]. Knowledge users for this study primarily include MHO/MOH working in LPHU across Canada and were engaged in developing the study design in each of three ways [9]. First, they were asked to identify specific areas that they would be most interested in learning from the research by co-creating the proposal and protocol. Their feedback was used to identify priority research questions for the project to address. Second, knowledge users were asked to provide feedback on draft survey questions with particular attention to comprehensiveness, clarity, cohesiveness, length, and the value of the information that would be gathered. Finally, the survey instrument was also piloted with three knowledge users and further improvements made to it based on their responses and feedback.

## Materials and methods

### Aim

**Fig 3:**
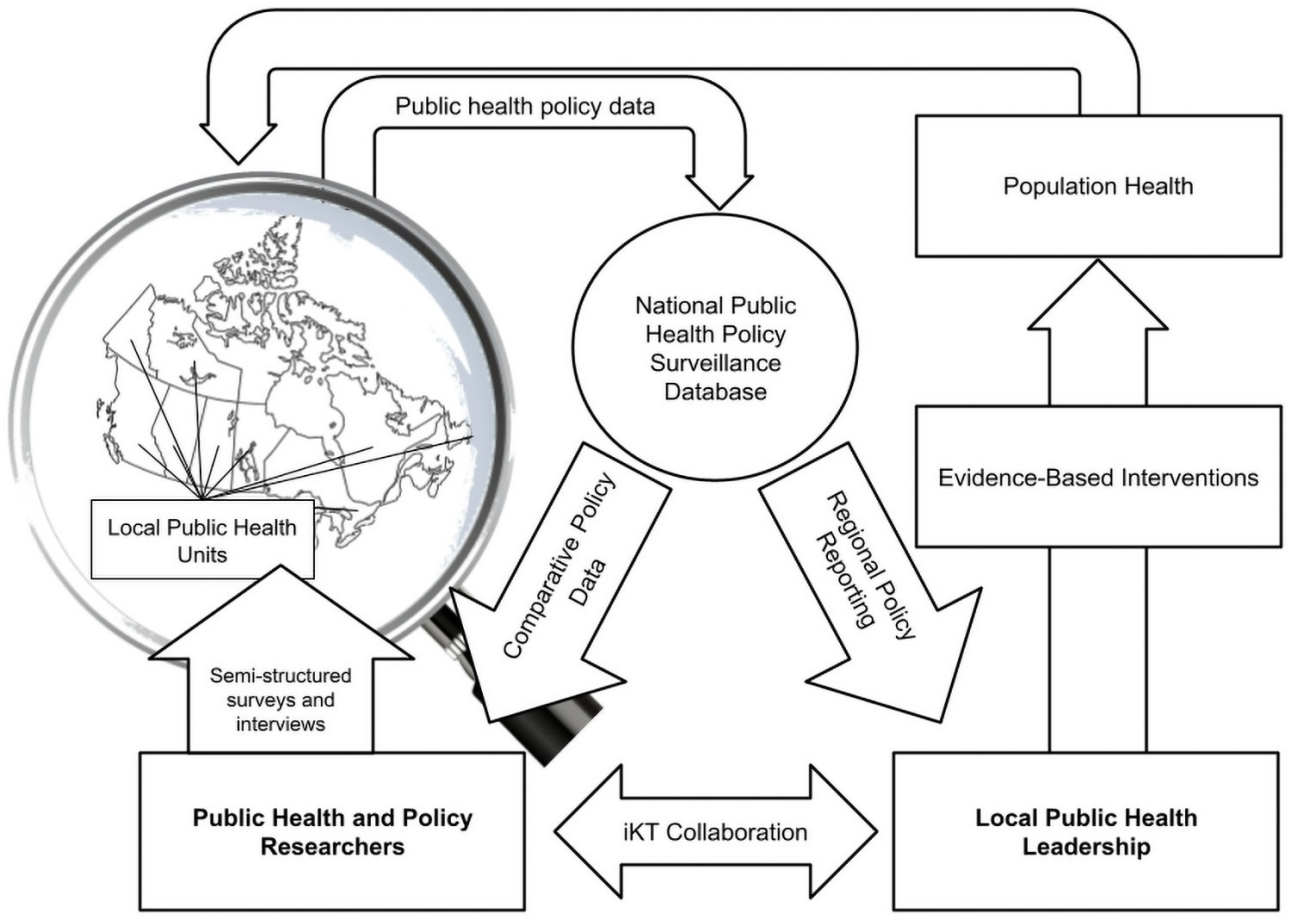
Policy surveillance model.

The aim of the research is to develop and implement a process that can routinely collect retrospective information on the local public health system response to the COVID-19 pandemic in Canada (Fig 3). An important outcome of this process will be to collect comparable data describing local public health operations during the pandemic, including governance structures, roles and responsibilities, functional scope, and capacity to enact public health programs and services. The study has been reviewed and approved by the University of Saskatchewan, Behavioural Research Ethics Board (Approval ID# 2409). The manuscript is a study protocol, therefore participant recruitment and obtaining consent has not begun. No results have been generated.

### Setting and data collection

The research will be led by researchers at the University of Saskatchewan and data collection will be carried out by the Canadian Hub for Applied and Social Research (CHASR; a research support service at the University of Saskatchewan). MHO/MOH will be interviewed by telephone because this will allow us to efficiently collect data from people working across the country. Also, based on our past experience surveying MHO/MOH, we expect that they will provide more fulsome responses than they would if we gathered this data using an online survey. The interview will follow a mixed methods sequential explanatory design [10]. This means that most of its sections contain a quantitative closed-ended question first, followed by a qualitative open-ended question. The purpose of this design is to strengthen quantitative results with explanations provided by qualitative data. Respondents will also be asked to volunteer any documents they may be able to provide that offers further information relating to their responses. The telephone survey has been designed to take no more than an hour. Each response will be transcribed by CHASR and shared with the research team for further processing and analysis.

### Study population

The target population for this study is MHO/MOHs and other public health leaders in LPHU and RPHU in Canada. In order to gather information on these units in Canada, we will administer a structured interview to MHO/MOHs and/or their designates (persons within their senior leadership teams who can respond to the survey on MHO/MOH’s behalf). Including designates is deemed critical to the study because MHO/MOH are extremely busy addressing COVID-19 and may not be able to participate personally but want their LPHU included in the study. The primary reasons for approaching MHO/MOHs (and/or designates) to collect data on LPHU is that they are a readily identifiable and distinct group of front-line local public health leaders whose roles and responsibilities are defined consistently across the country through legislation. As senior leaders within local public health, they possess the authority to speak knowledgeably about the local public health response to the pandemic. A list of all LPHU/RPHUs and corresponding MOHs in Canada was created by first, mining data from provincial/territorial and health authorities websites and second, verifying the list with research partners (UPHN and PHPC) for comprehensiveness (Table 1). In Table 1, provinces/territories and the RPHU and LPHU counts were created by authors based on web searches (e.g government and health authority websites) and verification with local experts (e.g. local MHO/MOH) and knowledge users, by population [11]. The research team used the expertise of the Urban Public Health Network (UPHN) and Public Health Physician of Canada (PHPC) to finalize the list of RPHU and LPHU in Canada.

### Recruitment

For this study, the unit of analysis are the RPHUs and LPHUs. We estimate that there are 126 RPHU and LPHU in Canada including 21 Indigenous-focused RPHU or LPHU (see Table 1). We will be inviting all 126 units to participate in this study. The objective is to recruit at least one MHO/MOH and/or their designates from each RPHU and LPHU; therefore, the study will employ a census approach to sampling RPHU and LPHU, as opposed to a random sample design. A response rate of at least 50% (n = 63) is thought to be achievable given the high interest of MHO/MOH and their participation in establishing aims and methods for the study.

Recruitment is a two step process. First, potential RPHU and LPHUs and their respective MHO/MOH will receive an invitation to participate in the study. UPHN and PHPC have agreed to forward the recruitment emails to their members. The recruitment email will include a Participant Information Sheet and Consent Form and MHO/MOH who choose to participate will be asked to complete a Contact Information Form via a link to an online survey. The Contact Form will include a question about consent, one of the two ways which consent will be obtained.

Once the Contact Form is submitted, project personnel will connect with the Canadian Hub for Applied and Social Research (CHASR) team and share the contact details of the respondents using email, a password-protected communication tool. The CHASR team will then reach out to the MHO/MOH and schedule a telephone interview by email. Audio-recorded, verbal consent will then be obtained prior to the telephone interview.

### Instrument design and development

Knowledge users in local public health were consulted to determine critical areas of concern and questions facing local public health systems leading into and during the COVID-19 pandemic. Five broad areas of concern were identified:

1. The role of MHO/MOH local public health experiences and insight in informing public health guidelines and measures and whether LPHU were able to tailor these to meet local needs.
2. The organization, structure, and governance of local public health throughout the country and the role of the MHO/MOH within these systems.
3. The extent of the collaborations and partnerships engaged in by local public health to respond to the pandemic.
4. The scope and quality of information and surveillance systems available to local public health to monitor and understand the pandemic as it unfolded at the local level.
5. The financial and human resources available to local public health to respond to the crisis.

In order to facilitate organization and analysis, these areas of concern were grouped into two broad categories: the first, those pertaining to *interventions*, and relating to the role of local MOH and local public health agencies in developing, overseeing, and administering efforts meant to manage and control the pandemic; and, the second, those pertaining to *capacities*, consisting of the latter four listed areas of concern, and relating to the resources local public health mobilize to enact and/or steer interventions.

The survey instrument was organized and developed around the five core sections, each reflecting a different one of the above listed areas of concern. Two sections were also added, first, at the beginning of the survey to confirm consent, and second, at the end of the survey to debrief and ask more general concluding questions about the most significant changes and barriers and facilitators of the local public health response to COVID-19 (See S1 File for survey instrument).

The section collecting information on the role of local public health in informing and, potentially, tailoring interventions was the most difficult to develop as there were no established methods for identifying and categorising interventions at the outset of the pandemic. A document review of six international and national monitoring and tracking initiatives that categorize public health interventions to address COVID-19 was completed [2–4, 12–14]. As many initiatives were focused on national or subnational levels, inclusion of categories of interventions was based upon the authors experience in determining the likelihood that LPHU’s would have roles and responsibilities with respect to each category of intervention. For example, interventions at the national level such as financial and economic interventions (e.g., provision of income support) were excluded as were interventions specific to the sub-national level such as those related to human resources (e.g., health workforce license reinstatement and reclassification). There was considerable overlap in categories of interventions from the documents reviewed (e.g., restrictions and regulations regarding closures and opening). If there was doubt about the relevance of an intervention at the LPHU level, it was included. A preliminary list of categories was prepared and an iterative process through piloting the interviews with Medical Officers of Health followed to collapse and/or adjust categories for interview questions.

Once categories of interventions to address COVID-19 were identified, they were grouped in the survey instrument under public health program and service areas that are familiar in the Canadian public health context [15]. Table 2 identifies three intervention categories within health protection and communicable disease control (i.e., restrictions and regulations, contact and case management, and vaccination) as well as various sub-categories of intervention. At the time of conducting the document review, vaccination was not a category in any initiatives and was added to the study protocol when the Government of Canada authorized the first vaccine for the prevention of COVID-19 [16]. Other program and service areas and categories from the document review are public health communication, emergency preparedness and response, and emergency social services (Table 2). Health equity was added as its own stand-alone question because reducing health inequities is a critical and overarching goal of effective public health systems and public health practice [17–20], and was emphasized by the Chief Medical Officer for Health with respect to the COVID-19 pandemic [1].

**Table 2:** Categories of LPHU interventions to address COVID-19.

Program/service area	Intervention category	Sub-category
Health protection & communicable disease control	Restrictions and regulations	Closures and openings: essential and non-essential businesses; education (pre-schools, schools, post-secondary, health care, recreation and culture, transportation and travel, tourism.
		Individual, family, and community behavior: mask use, physical distancing, family gatherings, mass indoor public gatherings, mass outdoor public gatherings.
	Contact and Case management	Assessment centres, test-track-trace, quarantine or isolation
	Vaccination	COVID-19
Health promotion, chronic disease & injury prevention	Health inequities	Target differential impacts of COVID-19 on vulnerable populations or specific social determinants of health equity.
Public health communication	Public awareness or education campaigns, media briefings	
Emergency preparedness & response	Emergency response plans, incident command centers	
Emergency social services	Domestic violence, sanitation measures, housing relocation, distancing strategies	

A preliminary version of the survey instrument was piloted with three MHO/MOH. Based on the responses given and additional consultation with knowledge users, the section of the survey on the role of local public health in informing and, potentially, tailoring interventions determined at provincial and federal levels was restructured to specifically centre on the role of local MHO/MOH in these activities. This was necessary because of the myriad arrangements of local public health systems present throughout the country and the fact that MHO/MOH were target respondents.

Broadly speaking, the sections designed to capture areas of concern 2–5 listed above can be said to focus on capturing different aspects of the *capacity* of local public health to respond to the pandemic. There are no agreed upon frameworks to study LPHU capacity, however, there are several frameworks that were found to be useful to articulate important domains, attributes or system elements [17, 21–27]. For the purposes of this study, the following system attributes or domains formed the foundation for examining LPHU capacity to address COVID-19: leadership and governance, partnerships and collaboration, financial and human resources, and surveillance and monitoring of COVID-19 [17]. Questions throughout the survey speak to these domains directly and are identified in Table 3.

**Table 3:** Domains of LPHU capacity to address COVID-19.

Capacity domain	Description
Leadership and governance	Governance structure of LPHU; Leadership/administrative structure; roles, responsibilities and relationships.
Partnerships and collaboration	Within the broader healthcare system and with sectors outside health (e.g., sectors, organizations, link to interventions, type of partnership, extent of collaboration).
Resources	Human, financial and technological (e.g., changes in resources and impact)
Information	Adequate monitoring and surveillance and monitoring capacity with respect to COVID-19 (e.g., differential impact of COVID-19 on certain populations; human resources to analyze and interpret data)

### Data processing and analysis

All transcribed interviews will be entered into NVivo software for coding and data analysis. The sequential explanatory design of the survey means that we will be able to extract both quantitative and qualitative findings. Data with respect to public health interventions will be coded to generate a) Boolean data on the role of the MHO/MOH in the development and implementation of measures and whether they were adapted to meet local needs, and b) qualitative data (descriptions or explanation of the quantitative responses). The structure of data gathered by capacity questions is more variable but it will undergo a similar process of quantitative and qualitative coding. A list of grouped and coded features of the local public health response to COVID-19 will be compiled for each LPHU and will be forwarded to interviewees for review (if they indicate that they want a draft). This list of coded local public health actions will form the primary output of the policy surveillance framework.

We will use unsupervised machine learning methods (specifically, cluster analysis techniques which are suitable for categorical data) to extract structured data on the different roles and approaches of local public health with respect to interventions in their response to COVID-19. We hypothesize that the identified clusters will correspond to overarching strategies used by different types of LPHUs in response to COVID-19 and will also be shaped by their capacities captured in other sections of the survey. This analysis will mirror the work of Mays et al. [21] who identified seven LPHU configurations in the United States. Another important precedent for our work is the Stringency Index developed by the Oxford COVID-19 Government Response Tracker team at the University of Oxford (2020) (OxCGRT) [4]. However, the OxCGRT measures and rates public health systems at the national-level, ignoring the heterogeneity in implementation of these measures at the local level. We hypothesize that overarching strategies at the LPHU-level in Canada will primarily be shaped by local-level operational constraints, governance arrangements and community characteristics. As such, the approaches that are identified by the cluster analysis will also provide an indirect measure of LPHU capacity. This approach has also been used for behavioural interventions to predict intervention effectiveness [28].

The Framework Method [29] of thematic analysis will also be applied to assess the qualitative answers to the open-ended questions that speak to public health policy features (program design, governance, capacity, financing, etc.). As the survey is designed to answer multiple policy-related research questions, a distinct analytic framework will be designed for each research question by multiple coders. Coding will involve an iterative process of familiarization, coding, framework development and deployment, interpretation and summarization. These codes will then be aggregated into themes based on the appropriate analytic framework for each research question, to explicate distinct public health policy phenomenon in question.

The two fundamental policy-related research questions that will guide this study’s analysis and shape the coding process are:

- How have the programs and services of the local public health system changed (stopped, adapted, enhanced) as a result of the pandemic?
- What have been the barriers and facilitators for Medical Officers of Health leadership in local public health systems in their response to the COVID-19 pandemic?

Data processing and analysis of the aforementioned policy-related research questions will be shaped by the study population’s response rate. Due to MHO/MOH’s role in the pandemic as front-line local public health professionals, they are constrained for time and resources. However, even if only half of RPHU/LPHU respond to the survey, we anticipate being able to answer these questions. This is a first-of-its-kind study to survey MHO/MOH’s working in LPHUs to gather comparable data on LPHU capacity to address COVID-19.

### Inclusion of federal, provincial and territorial public health policy contexts

Publicly available data sources will be merged with the local level data we capture that describes federal, provincial and territorial governance structures, and interventions (e.g. Canadian Institute for Health Information 2020) [30]. Also, key information on the population profiles of different local regions including the representation of different groups in local populations, including sex/gender, age group, ethnicity, citizenship status, labour force attachment, and income which can be constructed from long form Census records. This socio-demographic information will facilitate the interpretation of the roles and approaches of local public health identified by the quantitative and qualitative method and to consider whether determinants of approaches reside outside the health system.

## Discussion

### Ethical considerations and data sharing

Project personnel are sensitive to issues of anonymity and confidentiality and have limited the potential of individual participants to be identified. First, participants will be informed that data collected with respect to descriptions of public health interventions and COVID-19 surveillance data will be analyzed and reported by individual LPHU. By agreeing to participate, anonymity will not be completely protected because participants may be indirectly identified. We feel that concerns about anonymity and confidentiality are lessened because of the descriptive nature of this data and importantly the strong integrated knowledge translation approach to the research [9]. Additionally, the project will not be collecting and distributing information on the respondents themselves, but rather on their LPHU and the role of the MHO/MOH within them.

The study will generate three kinds of data, each having its own level of sensitivity and amenability to different kinds of analysis and dissemination. First, the transcribed interview data will be the richest but also the most sensitive. This data will not be shared beyond the research team and, per the University of Saskatchewan policies, will be destroyed within five-years after the end of the study. Second, the grouped and coded public health interventions and results of the cluster analysis, which will provide an overall comparable descriptive and quantitative portrait of LPHU activities across Canada, will be made available to researchers. Third, a stylized version of this dataset which is less suitable for analysis but will still provide an overview of LPHU efforts and capacity in Canada will be made publicly available. A team of knowledge users, which will include local MHO/MOH will be created to oversee which elements of the data will be shareable with other researchers and by what process, and which elements will be shareable with the public.

Participants will be informed via a Participant Information Sheet and Consent Form regarding these different ways of analyzing and reporting data.

### Output and implications of study

As a result of the complexities of conceptualizing and measuring local public health activities in Canada, much of the analysis of this project will actually be reflected in later iterations of our policy surveillance process itself. The authors will also report on the results of the meta-integrated knowledge translation efforts and how they gave rise to the areas of intervention and capacity that data are being collected on, and how this information will be grouped and coded to identify common and distinct public health activities across jurisdictions. The study will also report on the results of the cluster analysis to identify over-arching local public health approaches and how these reflect varying levels of capacity. As previously described, versions of these data and results will be made available to researchers and the public to advance research on and wider understanding of the local public health response to COVID-19 in Canada.

In the next stage of this research, our team will work with project partners and stakeholders to link LPHU interventions with COVID-19 outcomes. In order to advance this, we will identify available indicators of COVID-19 population health outcomes at the LPHU-level which will be effective for evaluating the impact of local public health responses and map these to the geographies associated with LPHU unit of analysis used by our policy surveillance framework. We anticipate that other researchers will want to work with our data to do similar research or inform their own research efforts to unpack what aspects of Canada’s COVID-19 response worked and what did not and what were the key drivers of these outcomes, for example, relating to governance, information, or resources.

As the first effort to systematically collect cross-country comparable data on the LPHU response to COVID-19, our study will provide an important foundation and precedent for the future development of policy surveillance processes and for the evaluation of LPHU-level data so that local public health in Canada can be better prepared for future crises. Indeed, the process developed for this study can be used as a tool in other research studies where the goal is to collect local surveillance level public health data on a regular basis. The integrated knowledge translation approach which we have adopted should advance both the pursuit of scientific knowledge of the Canadian public health system and provide LPHUs with information on the work of their peers that can inform their own practice.

## Supporting information

S1 File CIHR COVID-19 project: Survey instrument. (PDF)
