# Supplementary material for: Surveying the local public health response to COVID-19 in Canada: Study protocol
Source: PLoS One. 2021 Nov 18;16(11):e0259590. doi: 10.1371/journal.pone.0259590 (PMC8601475; doi:10.1371/journal.pone.0259590)
Supplement: S1 File — (PDF) [file pone.0259590.s001.pdf]

CIHR COVID-19 PROJECT: SURVEY INSTRUMENT

A. VERBAL CONSENT AND INTRODUCTION TO THE INTERVIEW

Thank you for your time and interest in this research.

A.1. Do I have your permission to start audio-recording to obtain your oral consent for the interview?

If no, stop the interview and ask interviewee how they would like to proceed.

If yes, start audio-recording and proceed.

A.2. Have you read the Participant Information Sheet and Consent Form and do you understand the purpose, procedures and the possible risks of the study?

If no, stop the interview and ask interviewee how they would like to proceed.

If yes

A.3. Do you consent to participate in this interview?

If no, stop the interview and ask interviewee how they would like to proceed.

If yes

By way of introduction, the focus of this research is on interventions and capacity to address COVID-19 at the local public health level. We are aware of public health interventions at the federal and provincial/territorial levels but, in this interview, we want to specifically learn about local public health level interventions and capacities.

There are many ways local public health is organized across the country. In this study we are interested in the lowest or most embedded level of public health administration of programs and services to a defined population. In some instances this may be called a local public health unit and in others the term "sub-provincial" may be used. Throughout this interview, we will refer to the "defined population" served by a local public health system as a "region." We acknowledge, however, that not all "regions" in this sense are contiguous, as is the case for several systems serving First Nations.

You have been recruited to the study because you are a Medical Officer of Health or a Medical Health Officer who has knowledge about local public health management of interventions and capacity to address COVID-19 in your region; or, you have been delegated by a Medical Health Officer to respond to this survey on their behalf.

Please note that in this survey, we will use the term "Medical Health Officer" to refer to both Medical Health Officers and Medical Officers of Health.

The interview is organized into 5 main sections:

The first section is focused on local public health leadership and governance, and this is followed by sections on local public health interventions that address COVID-19, partnerships and collaborations within and beyond the health system to address COVID-19, surveillance and monitoring of COVID-19 and population health, and finally, a section on financial and human resources to manage the pandemic.

B. LEADERSHIP AND GOVERNANCE

This first section of this survey is about local public health leadership and governance.

B.1. How would you describe the organization of local public health in your region in terms of leadership and governance? For example, please describe levels and positions that would appear in an organizational chart for local public health in your region or the reporting structures.

B.2.1. Please describe the roles and responsibilities of the Medical Health Officer within local public health in your region. For example, are they a manager, a leader, a consultant or some combination? Do they have public health staff who report to them?

B.2.2. What have been the main roles and responsibilities of the Medical Health Officer in addressing COVID-19?

B.3. Please describe how local public health in your region is situated in relation to the wider health system and other bodies of government. For example, what executive/portfolio or government agencies does the local Medical Health Officer in your region report to?

If respondent is an MOH, skip to Section C.

If respondent is a delegate

B.4. If you are a delegate, what is your role? What is the relationship between your role and the MOH? What is the relationship between your role and local public health operations?

C. PUBLIC HEALTH INTERVENTIONS

This second and largest section of the interview is about local public health interventions implemented over the course of the COVID-19 pandemic.

We use the term “Interventions” as a catch-all term to include all policies, programs, services, initiatives, measures, strategies, approaches, and laws and regulations implemented either directly or indirectly at the local level.

We want to study interventions from the perspective of local public health in your region and the role of the Medical Health Officer and staff who report to them.

We categorize interventions into the following 5 sections:

1. *Health protection and communicable disease control,*
2. *Public health communication,*
3. *Health equity-related interventions,*
4. *Emergency preparedness and response, and*
5. *Emergency social services.*

C.1. HEALTH PROTECTION AND COMMUNICABLE DISEASE CONTROL INTERVENTIONS TO ADDRESS COVID-19

First, I will ask questions about health protection and interventions specific to communicable disease prevention and control to address COVID-19. There are three areas of interest: restrictions and regulations, contact and case management, and vaccination.

Restrictions and regulations

C.1.1.1. Was the Medical Health Officer or staff who reported to them consulted on the development and implementation of restrictions and regulations specific to closures and openings in various private and public sectors in your region? By private and public sectors we mean: businesses, schools, health care offices, bars, etc. Please explain your answer.

C.1.1.2. Was the Medical Health Officer or staff who reported to them directly responsible for the inspection and enforcement of restrictions and regulations specific to closures and openings in various private and public sectors in your region? Please explain your answer.

C.1.1.3. In general, were restrictions and regulations specific to closures and openings in various private and public sectors adapted in any way to meet the specific needs or circumstances of your region? If yes, what kinds of adaptations were made?

This next question is about restrictions and regulations specific to individual, family and community behaviour over the course of the pandemic, like masking, physical distancing, restrictions on gatherings, etc.

C.1.2.1 Was the Medical Health Officer or staff who reported to them consulted on the development and implementation of restrictions and regulations specific to individual, family and community behaviour in your region? Please explain your answer.

C.1.2.2. Was the Medical Health Officer or staff who reported to them directly responsible for the inspection and enforcement of restrictions and regulations specific to individual, family and community behaviour in your region? Please explain your answer.

C.1.2.3. In general, were restrictions and regulations specific to individuals, family and community behaviour adapted in any way to meet the specific needs or circumstances of your region? If yes, what kinds of adaptations were made?

Contact and case management

Now we will be talking about contact and case management, so everything involving individual patients from contact tracing, testing, and isolation, to outbreak management across various settings.

C.1.3.1 Was the Medical Health Officer or staff who reported to them consulted on the development and implementation of contact and case management guidelines, human resources, and/or infrastructure in your region? Please explain your answer.

C.1.3.2 Was the Medical Health Officer or staff who reported to them directly responsible for the implementation of contact and case management work? Please explain your answer.

C.1.3.3 In general, were contact and case management practices adapted in any way to meet the specific needs or circumstances of your region? If yes, what kinds of adaptations were made?

Vaccination

We will now move onto questions regarding COVID-19 vaccinations:

C.1.4.1. Was the Medical Health Officer or staff who reported to them consulted on the development and implementation of COVID-19 vaccination program in your region? Please explain your answer.

C.1.4.2. Was the Medical Health Officer or staff who reported to them directly responsible for the implementation of the vaccination program in your region? Please explain your answer.

C.1.4.3. In general, were vaccination programs adapted in any way to meet the specific needs or circumstances of your region? If yes, what kinds of adaptations were made?

C.2. PUBLIC HEALTH COMMUNICATION

This next section shifts focus to public health communication strategies to address COVID-19:

C.2.1.1. Was the Medical Health Officer or staff who reported to them consulted on the development and implementation of public awareness or education campaigns around COVID-19 which aired in your region? Please explain your answer.

C.2.1.2. Was the Medical Health Officer or staff who reported to them directly responsible for the implementation of public awareness or education campaigns around COVID-19 which aired in your region? Please explain your answer.

C.2.1.3. In general, were public awareness or education campaigns adapted in any way to meet the specific needs or circumstances of your region? If yes, what kinds of adaptations were made?

C.2.2.1. Was the Medical Health Officer or staff who reported to them involved in talking to the media about COVID-19 in your region, provincially, or nationally? Please explain your answer. If they were not involved, can you share why not?

C.2.2.2. Prior to the onset of COVID-19, was the Medical Health Officer or staff who reported to them involved in communication with the media? Has this role changed since the onset of COVID-19?

C.3. EMERGENCY RESPONSE

This section is about emergency response to address COVID-19:

C.3.1.1. Was the Medical Health Officer or staff who reported to them consulted on the development and implementation of the emergency response to COVID-19 in your region? Please explain your answer.

C.3.1.2. What was the role of the Medical Health Officer or staff who reported to them in the implementation of the emergency response to COVID-19 in your region? Please explain your answer.

C.3.1.3. In general, was the emergency response to COVID-19 different from the responses to past emergencies in your region? Please explain your answer.

C.4. EMERGENCY SOCIAL SERVICES

In this section I will ask you about emergency social services that occurred during the COVID-19 pandemic, for example, initiatives involving housing, shelters, and food security.

C.4.1.1. Was the Medical Health Officer or staff who reported to them consulted on the development and implementation any emergency social services in your region? Please explain your answer.

C.4.1.2. Was the Medical Health Officer or staff who reported to them directly responsible for the implementation of any emergency social services in your region? Please explain your answer.

C.4.1.3. In general, were emergency social services adapted in any way to meet the specific needs or circumstances of your region? If yes, what kinds of adaptations were made?

C.5. HEALTH EQUITY

In this section I will ask you how health equity factored into the local response to COVID-19.

C.5.1.1. Has the Medical Health Officer or staff who reported to them consulted on explicitly addressing health inequities in the COVID-19 response in your region? Please explain your rating.

C.5.1.2. Was the Medical Health Officer or staff who reported to them directly responsible for the implementation of measures designed to explicitly address health inequities in the COVID-19 response in your region? Please explain your answer.

C.5.1.3. In general, was the COVID-19 response modified to address health inequities in your region? For instance, have interventions targeted differential impacts of COVID-19 on vulnerable populations? Or targeted specific social determinants of health? Please explain and provide examples.

D. PARTNERSHIPS AND COLLABORATION

In the following questions, we will ask you to report on the extent of partnerships and collaborations between local public health in your region and various local and regional service providers.

I will ask that you rate the extent of these partnerships and collaborations by selecting from one of the following three options: “to no extent,” “to some extent,” or “to a great extent.” Please let me know if you want me to reread this list at any time.

D.1.1 Please rate the extent to which local public in your region has partnered and collaborated <u>within the broad health care sector</u> to address COVID-19 with respect to the following:

D.1.1.1. Primary Care Clinics/Networks

D.1.1.3. Home care or community care

D.1.1.4. Hospitals

D.1.1.5. Long-term care

D.1.1.6. First Nations health organizations

D.1.1.7. Other local public health regions in your province or beyond

D.1.1.8. Provincial health department

D.1.1.9. Federal health departments

D.1.1.10. Non-profit, non-government and/or community-based health-related organizations

D.1.1.11. For-profit health business and industry

D.1.1.12. Health research organizations

D.1.2. Please describe the overall role of local public health in your region in partnering and collaborating within the broader health care sector to address COVID-19.

D.2.1. This next list includes potential partners to address COVID-19 outside the health care sector. Again, please rate the extent to which local public health in your region has partnered and collaborated with the following:

D.2.1.1. Other provincial departments beyond health (for example, transportation)

D.2.1.2. Other federal government departments beyond health

D.2.1.3. Municipal government departments (if local public health not embedded in municipal governance)

D.2.1.4. Other non-government, community-based organizations that do not primarily deliver health services

D.2.1.5. Education institutions including pre-schools, schools, and post-secondary institutions at the local, regional or provincial levels

D.2.1.6. Business and industry (for example, media partners)

D.2.1.7. Cultural and faith based organizations

D.2.2. Please describe the overall role of local public health in your region in partnering and collaborating outside the health care sector to address COVID-19.

E. SURVEILLANCE AND MONITORING

In this section of the interview I will ask you questions about monitoring and surveillance of local population health.

E.1.1. I will read a list of categories of health data. Please state yes or no as to whether the Medical Health Officer or staff who reported to them have had timely access to these types of data during the pandemic.

E.1.1.1. COVID-19 Testing results

E.1.1.2. Data regarding contact tracing for COVID-19

E.1.1.3. COVID-19 health outcomes (cases, deaths, hospitalization, etc.)

E.1.1.4. COVID-19 health outcomes stratified by sociodemographics (eg. race-based, immigrant status, income-level-based data)

E.1.1.5. Routine local public health outcomes (for example, childhood immunization coverage rates, STI rates, etc.)

E.1.2. Please describe the monitoring and surveillance technology or infrastructure that are used by local public health in your region to respond to COVID-19 (e.g., internal public health information systems, publicly available dashboards, etc.).

E.2. Has the Medical Health Officer or staff who reported to them had access to dedicated local analyst support to provide surveillance data during this pandemic? Has this access to analytic support changed compared to before the pandemic? Please explain your answer.

E.3.1. Do you or others in your office or unit have monitoring and surveillance data with respect to COVID-19 at the local level that you can share with us? For example: number of cases, tests, and tracing over the course of the pandemic.

If no (go to next section - F)

If Yes or Don’t know

E.3.2. Can we follow up with you via the email we have from your contact form?

If yes go to next question

If no

E.3.3. How would you like the research team to contact you?

F. RESOURCES

This is the second to last section of the interview and here I will ask you about the financial and human resources of local public health in your region to address COVID-19.

F.1. How did developments in the governance of local public health in your region prior to the pandemic impact the local response to the pandemic? Please explain your answer.

F.2. How did developments in the resourcing of local public health in your region prior to the pandemic impact the local response to the pandemic? Please explain your answer.

F.3. Have the financial resources of local public health in your region changed during the COVID-19 pandemic? If so, how? For example, have financial resources increased or decreased? Please explain your answer.

F.4. Has there been an extra need for human resources to address COVID-19 within local public health in your region and has this need been met? Have people been redeployed away from routine services? If so, in what areas and or for what interventions? Please explain and give examples if changes in the workforce have taken place.

F.5.1. This question will get at the impact of COVID-19 on routine public health programs and services. I will provide a list and please answer whether programs or services were stopped, adapted or enhanced, or whether programs or services in this area were not previously provided:

F.5.1.1. Health promotion programs

F.5.1.2. Routine environmental health inspection

F.5.1.3. Infant/Childhood immunization

F.5.1.4. School-based immunizations

F.5.1.5. Harm reduction services

F.5.1.6. Blood-borne and sexually transmitted infections programs

F.5.2. Are there other public health programs or services that were stopped, adapted or enhanced that we did not just explicitly ask you about? Please list them.

G. FINAL COMMENTS

This is the last section of the interview and here I ask five general questions about local efforts to address COVID-19 in your region.

G.1.1. What have been the most significant enabling or facilitating factors with respect to the local public health response to COVID-19 in your region?

G.2.1. What have been the most significant challenges or constraints with respect to the local public health response to COVID-19 in your region?

G.3.1. What have been the most significant changes to the local public health system over the course of the COVID-19 pandemic?

G.4.1. Have we missed any key areas or anything important regarding how local public health has responded to COVID-19 in your region? For example, are you surprised that something was missing in the interview?

G.5.1. Lastly, do you have existing documentation on how local public health in your region has addressed COVID-19 that you can share with the research team? For example, from an incident command management system, publicly available reports, or public health communications.

If No (go to conclusion)

If Yes or Don't know

G.5.2. The research team has your email and will follow up with you if that's OK?

This ends the interview and we want you to know that we greatly appreciate your time and interest in this research.

Thank you very much from the entire research team!
